# Evaluation of the Dacos 3.0 analyser

**DOI:** 10.1155/S1463924690000207

**Published:** 1990

**Authors:** Joan Farré Pons, Alba Alumá, Felipe Antoja, Carmen Biosca, María-Jesús Alsina, Román Galimany

**Affiliations:** 1 Servei d'Anàlisis Clíniques Centre d'Assistència Primàlria ‘Dr. Robert’ Plaça de la Medicina Badalona Barcelona s/n 08915 Spain; 2 Servei d'Anàlisis Clíniques Hospital ‘Germans Trías i Pujol’ Carretera de Canyet s/n 08916 Badalona Barcelona Spain

## Abstract

The selective multitest Coulter Dacos 3.0 analyser was evaluated according to the guidelines of the Comisión de Instrumentación de la Sociedad Española de Química Clínica and of the European Committee for Clinical Laboratory Standards.

The evaluation was performed in four steps: examination of the analytical units; evaluation of routine working; study of interferences; and assessment of practicability.

The evaluation included a photometric study. The inaccuracy is acceptable for 340 nm and 420 nm, and the imprecision at absorbances from 0.05 to 2.00 ranged from 0.06 to 0.28% at 340 nm and from 0.06 to 0.08% at 420 nm. The linearity showed some dispersion at low absorbance for PNP at 420 nm and the drift was negligible.

The imprecision of the pipette delivery system, the temperature control system and the washing system were satisfactory.

In routine work conditions, seven analytical methods were studied: glucose, creatinine, iron, total protein, AST, ALP and calcium. Within-run imprecision ranged, at low concentrations, from 0.9% (CV) for glucose, to 7.6% (CV) for iron; at medium concentrations, from 0.7% (CV) for total protein to 5.2% (CV) to creatinine; and at high concentrations, it ranged from 0.6% (CV) for glucose to 3.9% (CV) for ALP.

Between-run imprecision at low concentrations ranged from 1.4% (CV) for glucose to 15.1% (CV) for iron; at medium concentrations it ranged from 1.2% (CV) for protein to 6.7% (CV) for iron; and at high concentrations the range is from l.2for AST to 5.7% (CV) for iron.

No contamination was found in the sample carry-over study. Some contamination was found in the reagent carry-over study (total protein due to iron and calcium reagents). Relative inaccuracy is good for all the constituents assayed. Only LDH (high and low levels) and urate (low level) showed weak and negative interference caused by turbidity, and γ-GT (high level) and amylase, bilirubin and ALP (two levels) showed a negative interference caused by haemolysis.


Journal of Automatic Chemistry, Vol. 12, No. 4 (July–August 1990), pp. 153–162

### Evaluation of the Dacos 3.0 analyser

**Joan Farré Pons, Alba Alumá, Felipe Antoja, Carmen Biosca,† María-Jesús Alsina and Román Galimany†**

*Servei d'Anàlisis Clíniques, Centre d'Assistència Primària 'Dr. Robert', Plaça de la Medicina, s/n 08915 Badalona, Barcelona, Spain*

*The selective multitest Coulter Dacos 3.0 analyser was evaluated according to the guidelines of the Comisión de Instrumentación de la Sociedad Española de Química Clínica and of the European Committee for Clinical Laboratory Standards.*

*The evaluation was performed in four steps: examination of the analytical units; evaluation of routine working; study of interferences; and assessment of practicability.*

*The evaluation included a photometric study. The inaccuracy is acceptable for 340 nm and 420 nm, and the imprecision at absorbances from 0·05 to 2·00 ranged from 0·06 to 0·28% at 340 nm and from 0·06 to 0·08% at 420 nm. The linearity showed some dispersion at low absorbance for PNP at 420 nm and the drift was negligible.*

*The imprecision of the pipette delivery system, the temperature control system and the washing system were satisfactory.*

*In routine work conditions, seven analytical methods were studied: glucose, creatinine, iron, total protein, AST, ALP and calcium. Within-run imprecision ranged, at low concentrations, from 0·9% (CV) for glucose, to 7·6% (CV) for iron; at medium concentrations, from 0·7% (CV) for total protein to 5·2% (CV) to creatinine; and at high concentrations, it ranged from 0·6% (CV) for glucose to 3·9% (CV) for ALP.*

*Between-run imprecision at low concentrations ranged from 1·4% (CV) for glucose to 15·1% (CV) for iron; at medium concentrations it ranged from 1·2% (CV) for protein to 6·7% (CV) for iron; and at high concentrations the range is from 1·2 for AST to 5·7% (CV) for iron.*

*No contamination was found in the sample carry-over study. Some contamination was found in the reagent carry-over study (total protein due to iron and calcium reagents). Relative inaccuracy is good for all the constituents assayed. Only LDH (high and low levels) and urate (low level) showed weak and negative interference caused by turbidity, and γ-GT (high level) and amylase, bilirubin and ALP (two levels) showed a negative interference caused by haemolysis.*

### Introduction

The Dacos 3.0 analyser (Coulter Electronics Inc.) was evaluated according to the guidelines of the European Committee for Clinical Laboratory Standards (ECCLS) [1] and the protocol of the Comisión de Instrumentación de la Sociedad Española de Química (SEQC) [2].

The evaluation was performed in four steps: examination of the analytical units, evaluation in routine operation, study of turbidity and haemolysis interferences, and assessment of practicability.

The evaluation of the analytical unit included a photometric study: inaccuracy, imprecision, drift and linearity, as well as the imprecision of the pipette delivery system, the carry-over of both the sample and reagent delivery systems and the temperature control system.

Imprecision (within-run and between-run), carry-over and relative inaccuracy were studied in routine working conditions. Seven analytical methods: creatinine, protein, aspartate aminotransferase, glucose, alkaline phosphatase, iron (II + III) and calcium (II) were chosen in order to test nearly all of the performance criteria of the instrument. The relative inaccuracy was studied in comparison with the results obtained with the Technicon Chem 1 and Ultralab-Aurora analysers.

A study of interferences caused by haemolysis and turbidity was also made, according to the protocols of the Comission Validation de techniques de la Société Française de Biologie Clinique [3] and the International Union of Pure and Applied Chemistry [4].

The analyser's practicability was also evaluated by checking performance, analytical procedure control, maintenance and other aspects.

### Materials and methods

### *Analytical units*

#### *Instruments*

The Coulter DACOS 3.0 (Discrete Analyser with Continuous Optical Scanning) is manufactured by Coulter Electronics, Inc. The instrument is designed for determination of enzymes, substrates and therapeutic drugs. The routine procedure can be interrupted by stat samples at any time, returning afterwards to the original sequence.

The individual reagents are pipetted directly into the plastic reaction cuvette, incubated and read by a photometer with six fixed wavelengths and two optional wavelengths.

The sample pipettor allows a smooth adjustment to any volume between 2–20 µl. Subsequently, pipetting of two different reagents can be performed. The range of the reagent dispensing volume is 80–300 µl (reagent arm 1),

† Servei d'Anàlisis Clíniques, Hospital 'Germans Trías i Pujol', Carretera de Canyet, s/n 08916 Badalona, Barcelona, Spain.
Non-standard abbreviations used in this paper are: CD, Coulter Dart; TCN, Technicon; B, Behring; AST, aspartate aminotransferase (EC 2.5.1.2); ALP, alkaline phosphatase (EC 3.1.3.1); AMP, 2-amino-2-methyl-1-propanol buffer; PNP, *p*-nitrophenol; NADH, β-nicotinamide adenine dinucleotide, reduced form; NaOH, sodium hydroxide; TPTZ, tripiridyltriazine; c.l., confidence limits.

0142–0453/90 $3.00 © 1990 Taylor & Francis Ltd.

20–200 μl (reagent arm 2) and 20–200 μl (sample diluent delivery system).

The instrument is microprocessor-controlled. It enables the operator to have an overview of mechanics, electronics and chemistries at any time.

Mettler AC 100 Precision Balance;
Kontron-Uvikon 810 Spectrophotometer;
Fluka E/J Digital Thermometer.

*Reagents*

PNP 709·5 μmol/l (Sigma 104–8); NaOH (Merck 6498); from a solution of 0·36 mmol/l of PNP in NaOH (20 mmol/l) different concentrations were obtained by dilution; NADH disodium salt (Sigma N8129); Tris-(hydroxymethyl) methylamine (Merck 8382); all the other solutions were prepared from 1 mmol/l solution of NADH in Tris 80 mmol/l; Amido Black (Cromatest P722).

For comparison studies:

*Instrument:* Coulter Dacos 3.0 analyser

*Reagents*

Glucose (CD 7546860) (hexokinase); creatinine (CD 7546860) (Jaffé, without deproteinization); iron (B AU40T) (tripyridyltriazine); total protein (CD 7546781) (Biuret, Gordnall); AST (CD 7546051) (IFCC, modified); ALP (CD 7546781) (AMP); calcium (CD 7546058) (cresolphtaleine).

Technicon Chem 1 (Tarrytown, New York)

*Reagents*

Glucose (TCN-T01-1460-53) (hexokinase); creatinine (TCN-T01-1456-53) (Jaffé without deproteinization); AST (TCN-T01-1631-53) (IFCC); total protein (TCN-T01-1480-53) (Biuret, Gordnall); calcium (TCN-T01-1614-53) (cresolphtaleine); ALP (TCN-T01-1457-53) (AMP).

*Instrument*

Ultrolab-Aurora (Ultrolab, Sweden).

*Reagent*

Iron (B AU407) (TPTZ). For interference studies the reagent was Intralipid 20% (896449) (Fides).

### Evaluated parameters

The following parameters were studied during the evaluation of the instruments.

*Photometric inaccuracy*

Photometric inaccuracy was studied at 340 nm with a solution of disodium NADH (333 μmol/l) in Tris buffer (80 mmol/l), and at 420 nm with PNP solution (143·8 μmol/l) in NaOH (20 mmol/l). Dilutions were prepared manually. Up to three consecutive measurements were made for each absorbance, using the same cuvette.

Inaccuracy was calculated from the obtained values and the theoretical values calculated from the coefficient of molar absorptivity of NADH and PNP, and verified in a reference spectrophotometer.

*Photometric imprecision*

From solutions prepared as previously, 30 successive measurements were obtained in the same cuvette, and from these, the mean, standard deviation and coefficient of variation, at both 340 and 420 nm, were calculated.

*Photometric linearity*

Using serial dilutions prepared as before, three successive determinations were made for each absorbance, always in the same cuvette. Theoretical absorbances were calculated as described in the accuracy study.

*Photometric drift*

Photometric stability was studied over the first 30 min at intervals of 1 min, and over 8 h, at intervals of 1 h, at 550 nm, using a Black Amido solution of absorbance 0·590.

*Delivery systems' imprecision and reagent delivery systems' relative inaccuracy*

This study was made according to the following combinations: (1) sample and sample diluent delivery systems; (2) sample, sample diluent and reagent 1 delivery systems; (3) sample, sample diluent and reagent 2 delivery systems; (4) sample, sample diluent, reagent 1 and reagent 2 delivery systems. Different dispensing volumes were evaluated using PNP and NaOH solutions, at 420 nm. The coefficients of variation were calculated from 20 determinations.

*Carry-over*

This study was made according to the Broughton [5] and Bennet, modified [6], protocols, using PNP solutions at 420 nm. The sample delivery system and the reagent delivery systems were separately evaluated.

*Thermostatic system study*

This study was made by measuring the temperature of the reaction vessel containing destilled water using the probe of a digital thermometer.

Warm-up time was studied making readings every 20 s, until three consecutive readings with a deviation of ±0·1 °C were obtained.

Working temperature stability: 30 readings were made at 20 s intervals for 10 min, 45 min after power-on. The mean and coefficient of variation were calculated.

Room temperature influence: the protocol described for the working temperature stability study was applied at two different room temperatures (25·5 °C and 31·9 °C).

Temperature variation during the reaction: 45 min after power-on, the reaction warm-up time to reach 37 °C was studied using 123 μl of sample and diluent at 26·5 °C and

300 μl of reagent at 16 °C (distilled water was used as sample, diluent and reagent). The temperature was measured every 10 s, and this study was made in triplicate.

*Washing station study*

The effectiveness of the automatic washing of the reaction vessels was evaluated by reading the absorbances of distilled water before and after filling with a PNP solution of 1·6 absorbance at 420 nm. This study was made in 30 different reaction vessels.

*Between-reaction vessel imprecision*

The absorbances in 20 different reaction-vessels were measured, with blank correction, at 420 nm, filling each reaction-vessel with 400 μl of a 0·48 absorbance PNP solution. The mean and the coefficient of variation were calculated.

In the routine working evaluation, the parameters studied were:

*Imprecision*

Within the same run, 20 samples of control sera were tested at three levels, in order to study the within-run imprecision. To evaluate between-run imprecision, a further 20 samples were distributed in different runs.

*Reagent-related carry-over*

All combinations of method sequences were checked in order to study the reagent probe carry-over, using a pool of specimens in a pre-determined sequence run over three days. The carry-over effect measured was compared with twice the within-run imprecision of the method in question [1].

For example, the sequence employed to the glucose reagent (G) carry-over study was: G1–G2–G3–G4–G5–G6–IRON–G7–AST–G8–ALP–G9–CREATININE–G10–PROTEIN–G11–CALCIUM–G12–G13–G14–G15–G16–G17, and the carry-over caused by iron reagent was calculated according to:

$$(G7 - Gm) \times 100/Gm$$

$$Gm = (G2 + G3 + G4 + G5 + G6 + G13 + G14 + G15 + G16 + G17)/10.$$

*Sample-related carry-over*

Following a permutation order, two control samples with different concentrations were distributed along the sample disk. Three high specimens followed by three low specimens were processed and the carry-over was calculated according to the Broughton and Bennet, modified, protocols.

*Method comparison with patients' specimens*

One hundred fresh human sera were analysed (in different analytical series) covering the entire analytical range for each of the seven analytes, with the Coulter Dacos 3·0, and the comparison instruments Technicon Chem 1 and Ultrolab-Aurora. The statistical evaluation was done by a linear regression and correlation and non-parametric Passing–Bablok's regression [7–9].

*Interferences*

The effects of *in vitro* haemolysis and turbidity were evaluated on 16 constituents, according to the Commission for validation of methods of the Société Française de Biologie Clinique and IUPAC protocols.

These potential interferents were studied by overloading a human sera pool at different concentration levels of the analytes checked, with haemoglobin (up to 200 μmol/l) and lipid (up to 6 mmol/l of triglyceride). The assay value for each specimen was calculated as a percentage of the original (before overloading) concentration or activity.

*Practicability*

The practicability was evaluated in daily routine conditions of the authors' laboratory (350 samples/day with a mean of five tests/sample, without ISE module).

The following aspects were considered: system performance, environmental factors, maintenance, computer capabilities, operator's training, disadvantages and possible improvements, and failures of the system during the evaluation.

### Results and discussion

*Evaluation of the analytical modules*

*Photometric inaccuracy*

The photometric inaccuracy for NADH solution at 340 nm, expressed as percentage accuracy was −4·3% for 2·197 absorbance and −7·0% for 0·400 absorbance. For PNP solution at 420 nm it was 6·9% for 2·137 absorbance and 5·8% for 0·500 absorbance. The photometric inaccuracy is acceptable for both 340 and 405 nm (see table 1).

*Table 1. Photometric inaccuracy.*

| | Theoretical absorbance | Mean observed absorbance | Inaccuracy (%) |
|---|---|---|---|
| NADH (340 nm) | 0·400 | 0·372 | −7·0 |
| | 0·800 | 0·751 | −5·8 |
| | 1·200 | 1·137 | −5·2 |
| | 1·600 | 1·534 | −4·1 |
| | 1·997 | 1·923 | −3·7 |
| | 2·197 | 2·102 | −4·3 |
| PNP (420 nm) | 0·500 | 0·529 | 5·8 |
| | 1·000 | 1·069 | 6·9 |
| | 1·500 | 1·617 | 7·8 |
| | 2·000 | 2·137 | 6·9 |

*Photometric imprecision*

The coefficients of variation were always less than 0·30%. They ranged from 0·06 to 0·28% at 340 nm and from 0·06 to 0·08% at 420 nm (see table 2).

*Table 2. Photometric imprecision (N = 30).*

| | Mean absorbance | CV (%) |
|---|---|---|
| NADH (340 nm) | 0·039 | 0·28 |
| | 0·167 | 0·15 |
| | 0·411 | 0·08 |
| | 0·835 | 0·06 |
| | 1·706 | 0·07 |
| PNP (420 nm) | 0·062 | 0·08 |
| | 0·227 | 0·08 |
| | 0·529 | 0·08 |
| | 1·069 | 0·06 |
| | 2·137 | 0·07 |

*Photometric linearity*

The linearity obtained is acceptable for NADH at 340 nm ($r = 0{\cdot}99, y = -0{\cdot}01 + 0{\cdot}96x$ and for PNP at 420 nm ($r = 0{\cdot}99, y = -0{\cdot}05 + 1{\cdot}07x$). The results are shown in table 3. There is some deviation at low absorbances for PNP at 420 nm.

*Photometric drift*

For the photometric stability test, Black Amido solution was read at 550 nm for 30 min, using a solution with a mean absorbance of 0·590. The coefficient of variation obtained was 0·08%, and −0·16% absorbance deviation in this period. With the same solution read over 8 h, the coefficient of variation was 0·17%, and the absorbance deviation was −0·50% in this period. For both periods, the photometric drift (short-term and long-term) was negligible.

*Delivery systems' imprecision*

In both systems, sample and reagent delivery, the imprecision was less than 1·20% for all volumes and combinations evaluated (see table 4).

*Delivery systems' relative inaccuracy*

The delivery systems' relative inaccuracy was acceptable (see table 5).

*Carry-over*

The carry-over found in the sample delivery system was 0·005 (1·72%), and 0·197 (2·15%) in the reagent delivery systems, according to the Broughton and to the Bennet, modified, protocols respectively (see table 6). Carry-over is negligible in both systems.

*Temperature control*

Testing each 20 s, warm-up time to attain 37 °C was 43 min from the point at which the power was switched on (the results are shown in figure 1 at 5 min intervals).

Findings for attained temperature were as follows: main temperature, 37·4 °C; coefficient of variation, 0·134%, when the room temperature was 25·5 °C. Main temperature, 38·1 °C; coefficient of variation, 0·137%, when the room temperature was 31·9 °C. The working temperature seems to be affected by a high room temperature.

The warm-up time to reach the working temperature in the reaction vessel is 2 min (figure 2).

The temperature control system operates successfully as shown by the stability of the temperature, although the time to attain a stable temperature from power-up is long. The warm-up time to reach the working temperature in the reaction vessel is short.

*Washing station study*

The mean absorbance of 30 reaction vessels filled with distilled water before and after their filling up with a PNP solution was 0·10688 and 0·10784 respectively, with 0·00096 of mean absorbance increment. The range of individual vessel increments was between −0·004 and 0·003.

The automatic washing of the reaction vessels is satisfactory.

*Between-reaction vessel imprecision*

The coefficient of variation for a mean absorbance of 0·477 was 1·48%. (The spectrophotometric imprecision for this absorbance was 0·075%.)

The between-reaction vessel reading imprecision is good.

*Routine working evaluation*

*Imprecision*

Table 7 summarizes the studies of within-run and between-run imprecision. Within-run imprecision is acceptable for all the assayed analytes. Between-run imprecision is acceptable for all the assayed analytes, except for iron at low concentration.

*Reagent related carry-over*

In the study of the reagent related carry-over, no contamination was found, except a possible and small interference in the total protein by iron and calcium reagents. A more exhaustive study should be carried out (see table 8).

*Sample-related carry-over*

The results are shown in table 9. The means of the obtained K and C values are lower than 2·0% for all the analytes assayed. No significative contamination was found. The results are similar to those shown in table 6 (sample delivery systems) using aqueous sample solutions.

*Method comparison with patients' specimens*

The regression study for each of the analytes evaluated is shown in table 10. The results reflect good agreement with the comparison instruments. The coefficients of correlation ($r$) ranged from 0·897 (calcium) to 0·999 (glucose). Proportional and constant systematic differences ($p < 0{\cdot}05$) in glucose, AST and ALP and constant differences only for creatinine, were found in method comparison study according to Passing–Bablok regression.

*Table 3. Photometric linearity.*

| | NADH (340 nm) | | PNP (420 nm) | |
|---|---|---|---|---|
| Theoretical absorbance | Mean observed absorbance | Difference (%) | Mean observed absorbance | Difference (%) |
| 0·050 | 0·044 | −12·0 | 0·062 | 24·0 |
| 0·100 | 0·090 | −10·0 | 0·119 | 19·0 |
| 0·200 | 0·189 | −5·5 | 0·227 | 13·5 |
| 0·400 | 0·372 | −7·0 | 0·423 | 5·7 |
| 0·600 | 0·558 | −7·0 | 0·637 | 6·2 |
| 0·800 | 0·753 | −5·8 | 0·853 | 6·6 |
| 1·000 | 0·940 | −6·0 | 1·069 | 6·9 |
| 1·200 | 1·137 | −5·2 | 1·288 | 7·3 |
| 1·400 | 1·332 | −4·8 | 1·505 | 7·5 |
| 1·600 | 1·534 | −4·1 | 1·726 | 7·9 |
| 1·800 | 1·726 | −4·1 | 1·935 | 7·5 |
| 2·000 | 1·926 | −3·7 | 2·137 | 6·9 |

*Table 4. Imprecision of delivery systems.* N = *20, 420 nm.*

| Sample delivery system | | Reagent delivery systems | | | |
|---|---|---|---|---|---|
| Sample (μl) | Diluent (μl NaOH) | Reagent 1 (μl) | Reagent 2 (μl) | Mean absorbance | CV (%) |
| 3 (PNP) | 120 | — | — | 0·972 | 0·75 |
| 8 (PNP) | 40 | 80 (NaOH) | — | 1·205 | 0·81 |
| 3 (NaOH) | 120 | 100 (PNP) | — | 0·553 | 0·72 |
| 3 (NaOH) | 120 | — | 200 (PNP) | 0·758 | 1·05 |
| 3 (PNP) | 120 | 100 (NaOH) | — | 0·567 | 0·72 |
| 8 (PNP) | 40 | 80 (NaOH) | 200 (NaOH) | 0·487 | 1·17 |

*Table 5. Delivery system's relative inaccuracy.* N = *20, 420 nm.*

| | | | | Absorbance | | |
|---|---|---|---|---|---|---|
| Sample (μl PNP) | Diluent (μl NaOH) | Reagent 1 (μl NaOH) | Reagent 2 (μl NaOH) | Observed | Calculated | Inaccuracy (%) |
| 3 | 120 | 100 | — | 0·567 | 0·530 (A) | 6·89 |
| 8 | 40 | 80 | 200 | 0·487 | 0·470 (B) | 3·50 |

Calculated absorbance according to the dilution factor due to reagent volume knowing the sample + diluent (A) and the sample + diluent + reagent 1 (B) absorbances.

*Table 6. Delivery system's carry-over. PNP at 420 nm.*

Sequence: H1 –H2 –H3 –L1 –L2 –L3. H, high; L, low, $N = 10$

$$\text{Broughton: } K\,(\%) = \frac{L1 - L3}{H3 - L3} \times 100$$

$$\text{Bennet: } C\,(\%) = \frac{L1 - L3}{L3} \times 100$$

| | Mean absorbances | | | Carry-over | |
|---|---|---|---|---|---|
| | H3 | L1 | L3 | K (%) | C (%) |
| Sample delivery system | 1·680 | 0·00532 | 0·00523 | 0·005 | 1·72 |
| Reagent delivery system | 1·290 | 0·1111 | 0·1098 | 0·197 | 2·15 |

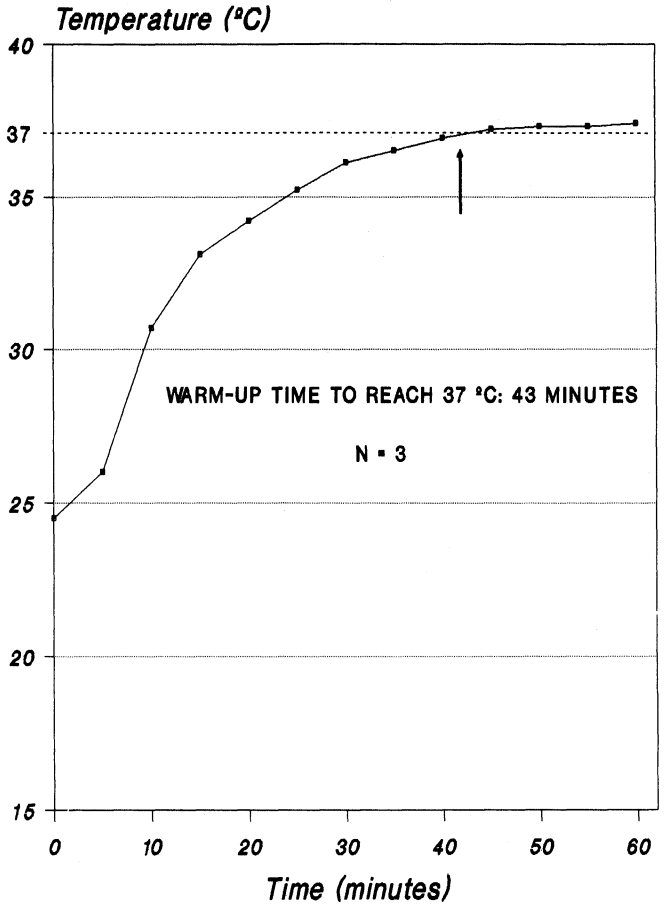

*Figure 1. Working temperature in reaction vessels. Warm-up time from power-on.*

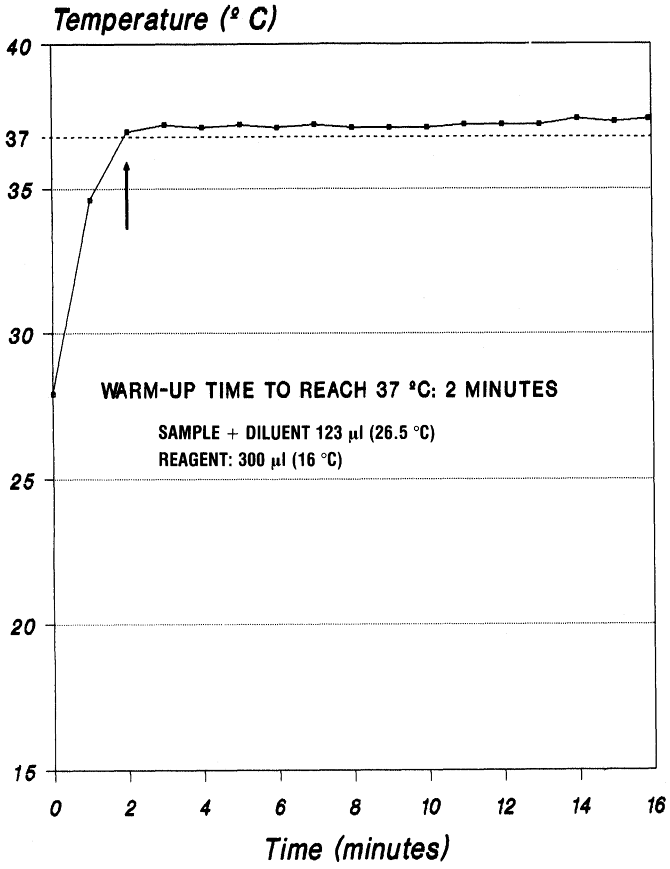

*Figure 2. Working temperature in reaction vessels. Variation during the reaction.*

*Interference*

A significant interference was considered to have occurred when the results obtained on the adulterated serum differed by at least three times the coefficient of variation (%) of the within-run imprecision for the level tested [4].

The interference study was made on AST, ALT, ALP, LDH, amylase, γ-GT, creatinine, urea, calcium, glucose, cholesterol, triglyceride, protein, phosphate, urate and total bilirubin at two concentration levels.

*Haemoglobin interference study*

At the maximum haemoglobin concentration of 200 µmol/l tested, a negative interference was observed in amylase at low concentration level 58 U/l (−34·0%) and high concentration level 533 U/l (−4·1%), total bilirubin at low concentration level 10·3 µmol/l (−33·0%) and high concentration level 39·3 µmol/l (−30·4%), ALP at low concentration level 75 U/l (−33·0%) and at high concentration level 582 U/l (−8·4%) and γ-GT at high concentration level 182 U/l (−5·5%) (see figure 3). No interference was noted for all the other analytes examined.

*Turbidity interference study*

At the maximum triglyceride concentration (6 mmol/l), a negative interference was observed with urate at 244 µmol/l (−4·8%), and with LDH at low concentration level 150 U/l (−6·6%) and high concentration level 382 U/l (−4·1%) (figure 4). No interference was noted for all the others analytes examined.

### Practicability

*Environmental factors*

The Dacos 3·0 requires adequate environmental control: 16–32 °C for room temperature with a variation less than 6 °C, 30–80% humidity without condensation, and 10 times/h air recycling.

It requires protection against bright light because of interference with the photometric system.

Water supply: maximum needed 40 l/h. NCCLS water requirement type II. Resistance 2MΩ/cm at 25 °C. Waste drain connection should be able to eliminate 55 l/h.

The system produces some noise due to delivery systems, washing station, compressor and printer.

*System performance*

Open system: different methods and reagents can be used. ISE and therapeutic drug monitoring are optional (not evaluated). Primary sample tube identification may be by bar-coding.

*Table 7. Within-run and between-run imprecision for concentrations and enzyme activities (mean ± SD) of some analytes measured with Coulter Dacos 3.0.*

| | | Within-run ($N$ = 20) $\bar{x}$ ± SD | CV (%) | Between-run ($N$ = 20) $\bar{x}$ ± SD | CV (%) |
|---|---|---|---|---|---|
| Glucose (mmol/l) | H | 17·0 ± 0·12 | 0·7 | 17·1 ± 0·23 | 1·3 |
| | M | 5·7 ± 0·08 | 1·4 | 5·8 ± 0·08 | 1·4 |
| | L | 2·9 ± 0·03 | 0·9 | 3·0 ± 0·04 | 1·4 |
| Creatinine (μmol/l) | H | 481 ± 7·7 | 1·6 | 498 ± 19·9 | 4·0 |
| | M | 87 ± 3·9 | 4·5 | 88 ± 5·1 | 5·9 |
| | L | 46 ± 2·8 | 6·0 | 49 ± 4·4 | 9·2 |
| Iron (μmol/l) | H | 38·0 ± 0·7 | 1·9 | 39·2 ± 2·2 | 5·7 |
| | M | 19·5 ± 0·6 | 3·0 | 20·4 ± 1·4 | 6·7 |
| | L | 5·5 ± 0·4 | 8·0 | 7·3 ± 1·0 | 15·0 |
| AST (UI/l) | H | 228·5 ± 2·0 | 0·9 | 222·3 ± 2·7 | 1·2 |
| | M | 37·8 ± 1·3 | 3·5 | 36·3 ± 1·8 | 5·0 |
| | L | 17·7 ± 1·0 | 6·1 | 18·9 ± 1·0 | 6·1 |
| ALP (UI/l) | H | 548·2 ± 13·7 | 2·5 | 540·8 ± 22·7 | 4·2 |
| | M | 112·0 ± 2·1 | 1·9 | 109·5 ± 3·3 | 3·0 |
| | L | 36·2 ± 1·3 | 3·6 | 35·6 ± 1·4 | 4·9 |
| Total protein (g/l) | H | 103·2 ± 0·8 | 0·8 | 102·5 ± 1·4 | 1·4 |
| | M | 75·0 ± 0·6 | 0·8 | 76·9 ± 0·8 | 1·1 |
| | L | 50·6 ± 0·5 | 1·0 | 51·7 ± 1·0 | 2·0 |
| Calcium (mmol/l) | H | 3·20 ± 0·02 | 0·8 | 3·36 ± 0·06 | 1·8 |
| | M | 2·45 ± 0·02 | 1·2 | 2·45 ± 0·05 | 2·2 |
| | L | 1·64 ± 0·01 | 1·2 | 1·65 ± 0·03 | 2·4 |

*Table 8. Reagent related carry-over.*

| Contaminator reagent $N$ = 3 | Observed difference in concentration of the constituent (%) Glucose | Calcium | Protein | Iron | Creatinine | AST | ALT |
|---|---|---|---|---|---|---|---|
| Glucose | — | −0·47 | 0·76 | −1·44 | −1·60 | 0·60 | −0·62 |
| Calcium | −0·70 | — | 1·64* | −0·34 | −1·60 | −5·40 | −0·62 |
| Protein | −1·03 | −0·13 | — | −1·05 | 1·60 | 0·66 | −0·60 |
| Iron | −0·70 | 0·20 | 1·64* | — | 1·60 | 0·80 | 1·65 |
| Creatinine | −1·03 | −0·13 | −0·13 | −1·05 | — | −3·20 | 0·52 |
| AST | 0·03 | −1·13 | 1·20 | −4·24 | −1·60 | — | 0·52 |
| ALP | −0·36 | −0·13 | 1·20 | 2·46 | 4·26 | −1·20 | — |

* Per cent difference more than twice the within-run imprecision. (CV for protein at medium concentration = 0·76%.)

*Table 9. Sample related carry-over.*

| | Concentration H3 | L1 | L3 | Carry-over K (%) | C (%) |
|---|---|---|---|---|---|
| Glucose (mmol/l) | 17·44 | 5·75 | 5·70 | 0·43 | 0·88 |
| Calcium (mmol/l) | 3·31 | 1·60 | 1·59 | 0·72 | 0·78 |
| Protein (g/l) | 94·80 | 49·70 | 50·10 | −0·89 | −0·80 |
| Iron (μmol/l) | 40·20 | 8·70 | 8·55 | 0·45 | 1·67 |
| Creatinine (μmol/l) | 472·10 | 85·75 | 85·75 | 0 | 0 |
| AST (UI/l) | 228·3 | 36·10 | 36·40 | −0·16 | −0·82 |
| ALP (UI/l) | 510·8 | 45·90 | 45·60 | 0·06 | 0·66 |

Observed sampling rate: 460 tests/h (without ISE).

It is possible to include 16 stat samples and to add 16 more samples while the sample plate (64 samples) is processing.

Reagent refrigeration system is at 15 ± 2 °C. Reagents are stable for a mean of 72 h.

Working temperature: 25, 30 or 37 °C.

Wavelength: 340, 420, 520, 575 and 630 nm (and two more optionals).

It is possible to keep the system in 'warm' state, without lamp consumption.

Messages and errors appear as direct screen information.

*Table 10. Relative inaccuracy. Results comparison of 100 human sera in (y) Coulter Dacos 3.0 versus (x) other analysers: (1) Technicon Chem 1 and (2) Ultrolab Aurora.*

| | Range | Coefficient of correlation (*r*) | Passing–Bablok's regression *b* (c.l. 95%) | *a* (c.l. 95%) |
|---|---|---|---|---|
| Glucose (mmol/l) (1) | 2·8–23·5 | 0·999 | 1·02 (1·01, 1·04)* | 0·21 (0·06, 0·28)* |
| Calcium (mmol/l) (1) | 1·67–4·68 | 0·897 | 1·08 (1·00, 1·18) | −0·08 (−0·28, 0·10) |
| Protein (g/l) (1) | 41·0–87·4 | 0·964 | 1·05 (0·99, 1·11) | −0·40 (−3·97, 2·63) |
| Iron (μmol/l) (2) | 2·68–27·7 | 0·913 | 0·97 (0·89, 1·05) | 2·88 (−2·92, 8·89) |
| Creatinine (μmol/l) (1) | 30–743 | 0·997 | 0·98 (0·93, 1·00) | 5·68 (2·85, 10·41)* |
| AST (UI/l) (1) | 8–3440 | 0·998 | 0·91 (0·88, 0·94)* | −2·33 (−3·39, −1·41)* |
| ALP (UI/l) (1) | 43–764 | 0·998 | 0·90 (0·89, 0·92)* | −2·36 (−3·61, −1·00)* |

$y = a + b\,x$; * = differences ($p < 0{\cdot}05$), constant (a), proportional (b).

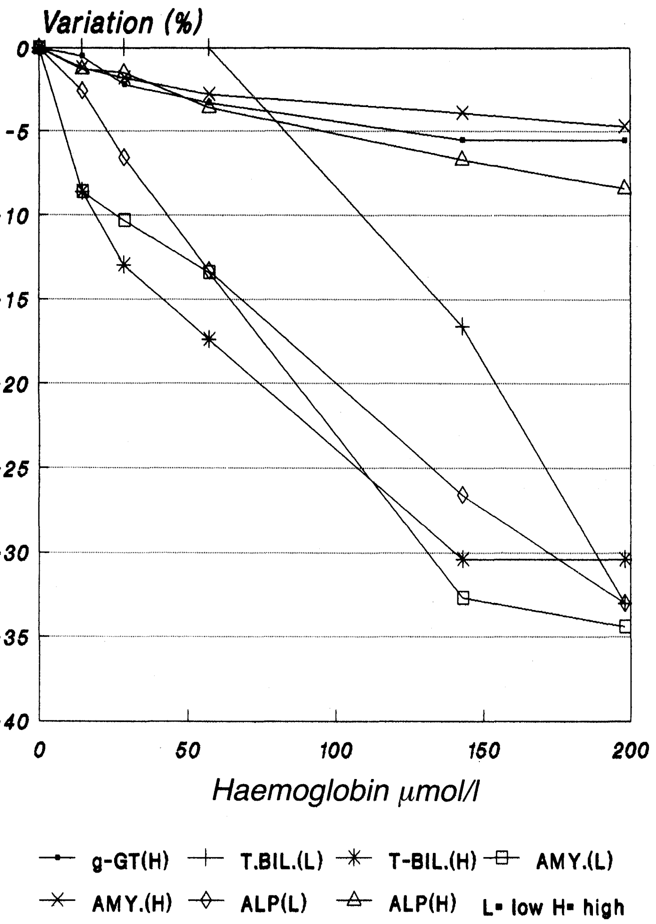

*Figure 3. Haemolysis interference.*

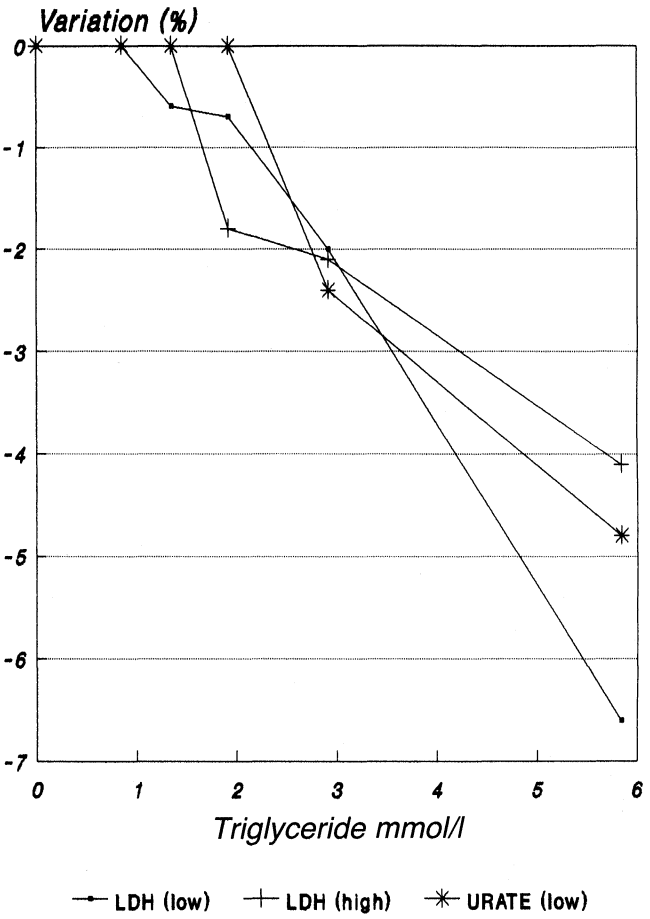

*Figure 4. Turbidity interference.*

*Calibration*

Optional and selective. Optional inclusion of controls. 16 Positions/plate capacity for calibrators or controls. Minimal frequency: weekly, and on new reagent lot.

*Fixed volumes per test:*

Sample: 2–20 μl.

Sample diluent: 20–200 μl.

Reagent: 80–300 μl.

Sample well capacity: 400 μl.

Calibrators and controls well capacity: 800 μl.

Residual volume: 50 μl.

Reaction vessel: 120–450 μl.

*Operational times*

Calibration and baselines: 11 min.

Switch on to 'ready' state: 15 min.

Switch on to first sample aspiration: 19 min.

'Off' state to 'warm' or 'ready' state: 10 min.

(The different states are displayed in the screen of the analyser. The instrument takes 43 min to reach working temperature in the reaction vessels.)

Reagents' preparation: 10 min.

Sample plate full programming (without reading manual bar-coded): 15 min.

Filling of samples and calibrators (without primary sample tube): 10 min.

Working list editing: 2 min.

Reaction process: 12 min 40 s for single reagent tests, and 9 min 52 s for two reagent tests.

*Computer capabilities*

Customized-order entry.

Fully flexible report format.

Search-and-sort data capabilities.

Includes the ability to store 3000 patient reports.

Real-time quality control, accumulates up to 61 working days. Levey-Jennings graphics.

Can be connected with other devices, to order entry, reception and editing results (not evaluated).

Two-way host computer interface (RS 232-C connection) (not evaluated).

*Alarm systems*

Information about system operation.

Information about control, calibration and patient results.

Efficacy of alarm systems has been checked and the results were satisfactory.

*Maintenance*

Daily: automatic washing (5 min).

Weekly: Cleaning of analyser and disk drive air filters. Sample plate cleaning.

Monthly: Control unit air filter cleaning. Washing station cleaning. Thermostatic system bath water change. Diluent container cleaning. Changing reaction vessels.

Every 15 000 cycles: Sample syringe change. Diluent syringe change. Diluent container cleaning.

Every 30 000 cycles: Reagent syringes change.

*Personnel*

Direct personnel: one technician for the analytical work.

Indirect personnel: one technician for sample preparation (if a primary blood sample tube is not available). One secretary (if on-line connection to the main computer is not available).

*Operator's training*

Routine operator: five days.

For complete knowledge of the system: 15 days.

*Operator's manuals*

Three volumes with exhaustive description of the system, including two different alarm codes indicating suggested solutions to any problems.

*System failures during the evaluation*

During the four months' evaluation:

*Short-term*

Fault in the lighting of the spectrophotometer lamp (this resolved itself).

Falling absorbance readings with the 420 nm filter (this was solved by changing the corresponding electronic circuit).

(These incidents were attributed to the transport of the equipment.)

*Medium-term*

Increasing aleatory error in the techniques using a high sample volume (solved by fixing the position of the sample pipette more accurately).

Isolated errors in pipette arm 2 when measuring the reagent volume (solved by priming).

### Disadvantages and possible improvements

Where the water quality is poor, the life of the deionizing columns can be short, due to the high consumption of deionized water by the analyser.

Environmental control is necessary. The analyser generates enough heat to increase the room temperature.

It would be helpful to incorporate the patients' daily means and the Westgard algorithms in the quality-control program.

It would be useful to be able to detect errors in the sample pipette position when delivering into the reaction vessel.

A facility for automatic starting should be made available.

The warm-up time to reach the working temperature in the reaction vessel should be shorter.

### References

1. European Committee for Clinical Laboratory Standards. Guidelines for the evaluation of analysers in Clinical Chemistry, *ECCLS Document*, Vol. 3, No. 2 (June 1986).

2. Comissión de Instrumentación del Comité Científico de la Sociedad Española de Química Clínica. Protocolo de Evaluación de analizadores automáticos: evaluación de los módulos analíticos, Doc. E, ver. 3; Bol. Inf. SEQC (1986), no. 34, 3.
3. Société Française de Biologie Clinique. Comission Validation de techniques', Protocole de validation de techniques. Document B, stade 3. *Annales de biologie clinique*, **44** (1986), 716.
4. International Union of Pure and Applied Chemistry, *Pure and Applied Chemistry*, **61** (1989), 91.
5. Broughton, P., *Journal of Automatic Chemistry*, **6** (1984), 94.
6. Bennet, A., Gastelmann, D., Mason, J. I., and Owen, J. A., *Clinica Chimica Acta*, **29** (1970), 161.
7. Passing, H. and Bablok, W., *Journal of Clinical Chemistry and Clinical Biochemistry*, **21** (1983), 709.
8. Passing, H. and Bablok, W., *Journal of Clinical Chemistry and Clinical Biochemistry*, **22** (1984), 431.
9. Bablok, W., Passing, H., Bender, R., and Schneider, B., *Journal of Clinical Chemistry and Clinical Biochemistry*, **26** (1988), 783.

